# 

**Published:** 1880-06

**Authors:** 


					﻿JUNE, 1880.
TAVENTY^SEVENTH YEA1Ì.
HALL’S
J ournal of Health.
E. H. GIBBS, A.M., M.D., Editor,
No: 141 EIGHTH STREET (near Broadway) NEW YORK.
Terms, $1.50 a Year, or $1.00 for Eight Months. Postage paid.
Entered as Second-Class matter in the Post-office in New York,
				

